# Beneficial Changes in Rat Vascular Endocannabinoid System in Primary Hypertension and under Treatment with Chronic Inhibition of Fatty Acid Amide Hydrolase by URB597

**DOI:** 10.3390/ijms22094833

**Published:** 2021-05-02

**Authors:** Marta Baranowska-Kuczko, Hanna Kozłowska, Monika Kloza, Ewa Harasim-Symbor, Michał Biernacki, Irena Kasacka, Barbara Malinowska

**Affiliations:** 1 Department of Experimental Physiology and Pathophysiology, Medical University of Białystok, ul. Mickiewicza 2A, 15-222 Białystok, Poland; hkozl@umb.edu.pl (H.K.); monika.kloza@umb.edu.pl (M.K.); bmalin@umb.edu.pl (B.M.); 2 Department of Clinical Pharmacy, Medical University of Białystok, ul. Mickiewicza 2A, 15-222 Białystok, Poland; 3 Department of Physiology, Medical University of Białystok, ul. Mickiewicza 2C, 15-222 Białystok, Poland; eharasim@umb.edu.pl; 4 Department of Analytical Chemistry, Medical University of Białystok, ul. Mickiewicza 2D, 15-222 Białystok, Poland; michal.biernacki@umb.edu.pl; 5 Department of Histology and Cytophysiology, Medical University of Białystok, ul. Mickiewicza 2C, 15-222 Białystok, Poland; kasacka@umb.edu.pl

**Keywords:** FAAH inhibitor, URB597, SHR, endocannabinoids, cannabinoid CB_1_ receptor

## Abstract

Our study aimed to examine the effects of hypertension and the chronic administration of the fatty acid amide hydrolase (FAAH) inhibitor URB597 on vascular function and the endocannabinoid system in spontaneously hypertensive rats (SHR). Functional studies were performed on small mesenteric G3 arteries (sMA) and aortas isolated from SHR and normotensive Wistar Kyoto rats (WKY) treated with URB597 (1 mg/kg; twice daily for 14 days). In the aortas and sMA of SHR, endocannabinoid levels and cannabinoid CB_1_ receptor (CB_1_R) expression were elevated. The CB_1_R antagonist AM251 diminished the methanandamide-evoked relaxation only in the sMA of SHR and enhanced the vasoconstriction induced by phenylephrine and the thromboxane analog U46619 in sMA in SHR and WKY. In the sMA of SHR, URB597 elevated anandamide levels, improved the endothelium-dependent vasorelaxation to acetylcholine, and in the presence of AM251 reduced the vasoconstriction to phenylephrine and enhanced the vasodilatation to methanandamide, and tended to reduce hypertrophy. In the aortas, URB597 elevated endocannabinoid levels improved the endothelium-dependent vasorelaxation to acetylcholine and decreased CB_1_R expression. Our study showed that hypertension and chronic administration of URB597 caused local, resistance artery-specific beneficial alterations in the vascular endocannabinoid system, which may bring further advantages for therapeutic application of pharmacological inhibition of FAAH.

## 1. Introduction

**Figure 1:**
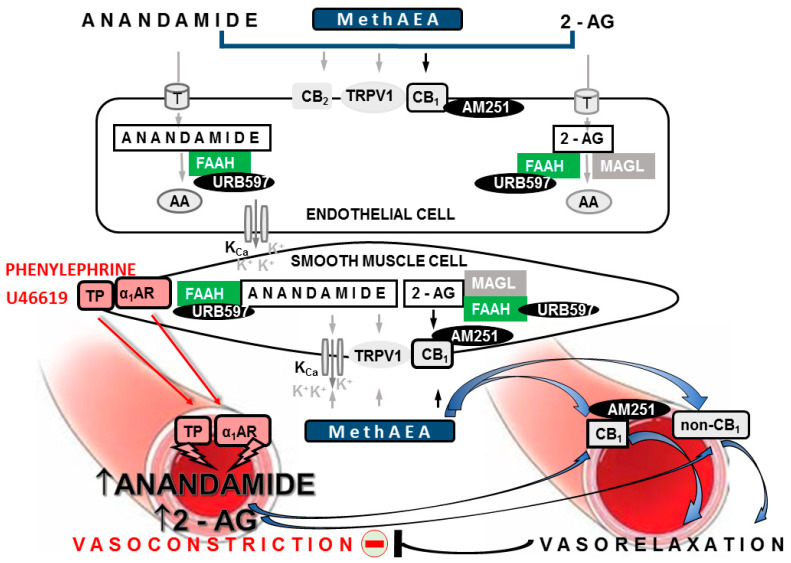
Summary of the mechanisms of the vasodilatory effects of endocannabinoids and their interaction with chosen vasoconstrictors in the resistance vasculature. The mesenteric arteries are used as an example. Endocannabinoids anandamide and 2-arachidonoylglycerol (2-AG), as well as methanandamide (MethAEA, synthetic stable analog of anandamide), induce relaxation of the mesenteric arteries via CB_1_-dependent and CB_1_-independent mechanisms (non-CB_1_). CB_1_ receptors are located in smooth muscle or endothelial cells. Non-CB_1_-mediated pathways include CB_2_ receptors, transient receptor potential vanilloid type-1 (TRPV1) and others. Stimulation of CB_1_ and non-CB_1_ pathways, in turn, may result in the activation of calcium-dependent potassium channels (K_Ca_) and hyperpolarization of smooth muscle cells. In addition, anandamide and 2-AG are metabolized to arachidonic acid (AA) via fatty acid amide hydrolase (FAAH) and monoacylglycerol lipase (MAGL), respectively. AA may be further converted into either vasodilator or vasoconstrictor eicosanoids (reviewed in [1,3]). Vasoconstrictors (e.g., agonists of α_1_-adrenergic receptors, phenylephrine or thromboxane TP receptors, thromboxane A_2_ analog—U46619) in addition to their direct contractile effects, indirectly mediate the rapid biosynthesis of endocannabinoids. Endocannabinoids may then activate the CB_1_- and non-CB_1_-dependent vasodilatory effects. The above-mentioned endocannabinoid negative feedback leads to the reduction of the agonist-induced vasoconstriction. The components of the endocannabinoid system and drugs that were considered in this study are marked in black frames. AM251—the antagonist of CB_1_ receptors; URB597—the FAAH inhibitor; T—endocannabinoid membrane transporter; black and gray arrows designate CB_1_- and non-CB_1_-mediated receptor changes, respectively; the red minus sign indicates reduction.

Hypertension is a devastating disease, affecting 20–50% of the world’s population. The endocannabinoid system is overactivated in hypertension (for reviews, see [1,2,3]). It consists of endocannabinoids, such as anandamide and 2-arachidonoyl glycerol (2-AG), the enzymes responsible for their synthesis (for details, see 2) and degradation—fatty acid amide hydrolase (FAAH) and monoacylglycerol lipase (MAGL), respectively as well as cannabinoid CB_1_ and CB_2,_ receptors. Endocannabinoids may also act in non-CB_1_/CB_2_ receptor manner, i.e., activating transient channel vanilloid type 1 (TRPV1), calcium-dependent potassium channels (K_Ca_) or via vasoconstrictor (i.e., thromboxanes) and vasodilator (i.e., prostaglandin E_2_, prostacyclin) arachidonic acid breakdown products derived from endocannabinoids (Figure 1; for reviews, see [1,2,3]).

The endocannabinoid system has been demonstrated to play—at least locally—a protective role in the vasculature. First, in rats with primary hypertension (spontaneously hypertensive; SHR) and in different models of secondary hypertension, cannabinoids (e.g., anandamide and its stable analog methanandamide; MethAEA) exerted stronger vasodilatory and hypotensive effects than in normotensive controls. Moreover, inhibitors of fatty acid amide hydrolase (FAAH; URB597 and AM3506), an enzyme responsible for endocannabinoid degradation, reduced blood pressure, which has not been observed in normotension [4,5,6,7,8,9], as reviewed by [1,10]. The above depressor effects have been demonstrated to be dependent on peripherally located neuronal and non-neuronal vascular cannabinoid CB_1_ receptors. (1) The enhancement of the potential protective effect of CB_1_ receptors, mediating the presynaptic inhibition of noradrenaline release from the sympathetic nerve endings innervating resistance vessels of pithed rats, was noticed in 11-deoxycorticosterone acetate (DOCA)-salt hypertension [11], but not in SHR [12]. (2) The bolus injection of the CB_1_ receptor antagonists rimonabant or AM251 increased blood pressure and myocardial contraction in SHR and rats with angiotensin II–vasopressin-induced hypertension [6]. (3) The CB_1_ receptors have been demonstrated to play a role in the enhanced vasorelaxation of the mesenteric G3 arteries of the DOCA-salt model [4,5] and thoracic aortas of two-kidney one-clip (2K1C) [13] rats induced by MethAEA, cannabidiol, and anandamide, respectively, as well as in the attenuated vasorelaxant response to cannabidiol in SHR [5]. They did not mediate the respective vasorelaxation in control littermates. (4) The upregulation of CB_1_ receptors at the protein and/or gene level has been noted in rat mesenteric G3 arteries in DOCA-salt [4] and SHR [5] and in the aortic endothelium of SHR [6] and 2K1C [13]. (5) Protective CB_1_ receptor-mediated vasorelaxation is induced by endocannabinoids released upon stimulation by various vasoconstrictors (e.g., an analog of thromboxane A_2_, U46619; phenylephrine; and angiotensin II) in the systemic (i.e., aortas, cerebral, resistance gracilis, and coronary arteries; [14,15,16,17]) and pulmonary circulations [18,19] (Figure 1). FAAH inhibitors have been suggested as a promising target for antihypertensive drugs, as they enhance endocannabinoid signaling [1,2]. We have previously reported that the administration of the FAAH inhibitor URB597 (1 mg/kg, twice daily) for 14 days decreased blood pressure in DOCA-salt rats, but not in SHR [4,20]. The URB597-induced reduction in blood pressure in DOCA-salt partially correlated with the protective role of cannabinoid CB_1_ receptors and with structural and functional changes in conductance and resistance vessels [4]. Moreover, the vasorelaxant effect of MethAEA in small mesenteric G3 arteries was mediated via TRPV1 in both normo- and hypertensive animals, either untreated or treated with URB597 [4].

Vascular dysfunction remains the leading cause of cardiovascular morbidity and premature death in the hypertensive population [3]. Importantly, the local beneficial effects of some compounds may not be visible as a clear hypotensive response. For example, the chronic administration of the phytocannabinoid cannabidiol reduced the carbachol-induced increases in coronary perfusion pressure in the hearts of hypertensive models (DOCA-salt and SHR) but failed to modify blood pressure in these animals [21]. Considering the above, the aim of our study was to explore the potential protective role of locally overactivated endocannabinoids and vascular cannabinoid CB_1_ receptors in developing functional and structural changes in resistance and conductance arteries in SHR chronically treated with URB597.

## 2. Results

### 2.1. General

As described previously [20], before treatment with the first dose of URB597 (or its vehicle), systolic blood pressure (SBP) was about 70% higher in SHR than in age-matched WKY (183 ± 7 mmHg, *n* = 10, vs. 104 ± 8, *n* = 10; *p* < 0.05). Chronic URB597 treatment did not change SBP in SHR (182 ± 7 mmHg, *n* = 10) or in WKY (93 ± 5 mmHg; *n* = 10). The mean tensions induced (in mN) by 120 mM KCl were similar between the WKY, WKY + URB597, SHR, and SHR + URB597 groups (mesenteric G3 arteries: 10.3 ± 0.6, *n* = 24; 9.5 ± 0.9, *n* = 24; 11.1 ± 1.8, *n* = 24; 10.6 ± 1.1, *n* = 24; aortas: 10.8 ± 1.0, *n* = 28; 11.5 ± 0.6, *n* = 28; 10.6 ± 0.8, *n* = 28; 9.6 ± 1.0, *n* = 28). Similarly, in all the experimental groups (i.e., WKY, WKY + URB, SHR, and SHR + URB), the phenylephrine-induced vasocontraction in small mesenteric G3 arteries (12.2 ± 2.5, *n* = 32; 10.9 ± 1.8, *n* = 32; 13.2 ± 2.0, *n* = 28; 11.9 ± 1.9, *n* = 28) and aortas (6.8 ± 0.5, *n* = 12; 7.4 ± 0.6, *n* = 12; 6.4 ± 0.3, *n* = 12; 6.8 ± 0.5, *n* = 12) were comparable. No significant effects of the vehicle for MethAEA and AM251 on vessel function were observed.

### 2.2. Influence of Hypertension; An Antagonist of the CB_1_ Receptor, AM251; and Chronic Administration of URB597 on Vasoconstrictor Responses to Phenylephrine and U46619

To assess the vascular contractile function, endothelium-intact mesenteric G3 arteries and aortas were exposed to phenylephrine: 0.01–30 µM and 0.001–30 µM and thromboxane analog U46619 0.001–3 µM and 0.0001–0.3 µM, respectively.

**Figure 2:**
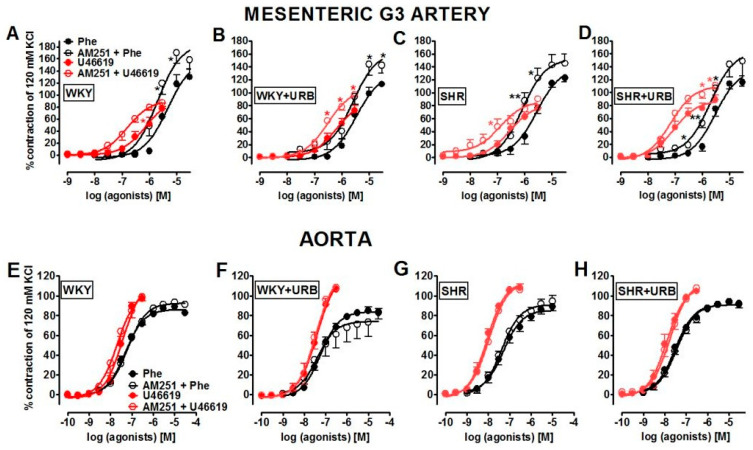
Influence of AM251 (1 µM) on the vasoconstrictive effects of phenylephrine (Phe, black circles) and U46619 (red circles) in the mesenteric G3 arteries (**A**–**D**) and aorta (**E**–**H**) from normotensive control (WKY; **A**,**E**) and URB597-treated (WKY + URB; B, F) rats, or hypertensive (SHR; **C**,**G**) and URB597-treated (SHR + URB; **D**,**H**) rats. URB597 at 1 mg/kg or its vehicle was injected intraperitoneally every 12 h for 14 days. Contractile responses are shown as percentages of the reference response to KCl. Mean ± SEM of *n* = 6–7 tissues for each curve. * *p* < 0.05 and ** *p* < 0.01 compared to the * WKY, as determined by Student’s *t-*tests for unpaired data. In a few cases, the SEM is smaller than or equal to the size of the symbols. See Table 1 and Table 2 for statistical analysis**.**

The α_1_-adrenoceptor agonist, phenylephrine, and thromboxane analog U46619 induced a concentration-dependent contraction of the rat mesenteric G3 arteries and aortas (Figure 2). Compared to WKY, SHR showed nearly a 2-fold increase in the vasoconstrictor responses to phenylephrine in mesenteric G3 arteries but not in the aortas. Simultaneously, the concentration–response curves (CRCs) for U46619 were shifted to the left by factors of ~2.5 and ~5 in small mesenteric G3 arteries and aortas, respectively. For the pEC_50_ and maximum extent of relaxation (R_max_) values, see Table 1; Table 2.

To understand whether the normal endocannabinoid tone controls vasoconstrictive response in control and hypertensive animals, we examined concentration-dependent contraction of mesenteric G3 arteries and aortas stimulated by phenylephrine and U46619 in the presence of the CB_1_ receptor antagonist, AM251 that antagonizes endocannabinoid signaling. The vasoconstrictor responses for phenylephrine and U46619 in the mesenteric G3 arteries of normo- and hypertensive rats (but not in aortas) were sensitive to the CB_1_ receptor antagonist AM251 (1 µM). The CRCs for both agonists were shifted to the left in the presence of AM251. In normotensive rats, CRCs were shifted by 2.5 and 5 factors, respectively, whereas in hypertensive animals, the shift factor was 2.5 in both cases. Additionally, a trend towards increased the maximal contraction mediated by U46619 and no change in the maximal response in phenylephrine-induced contraction were noticed. For the pEC_50_ and R_max_ values, see Table 1 and Table 2.

Subsequently, we examined whether URB597-mediated augmentation of endocannabinoid levels influenced the local contractile function of arteries. In this case, particular attention was paid to the role of the CB_1_ receptor in this process.

Interestingly, chronic URB597 administration enhanced the potency, but not efficacy, of U46619 only in the mesenteric G3 arteries of SHR and attenuated the vasoconstrictor response to phenylephrine in the presence of AM251 (Figure 2D). It did not change the vascular responses to both vasoconstrictors in the aortas of normo- and hypertensive rats (for the respective pEC_50_ and R_max_ values, see Table 1 and Table 2; Figure 2). Moreover, the blockade of CB_1_ receptors with AM251 in the mesenteric G3 arteries of rats treated with URB597 enhanced the vasoconstrictive response to U46619 in WKY (in both potency and efficacy) and in SHR (efficacy). For the pEC_50_ and R_max_ values, see Table 1 and Table 2.

### 2.3. Influence of Hypertension and Chronic Administration of URB597 on Vasodilatory Effects of Acetylcholine and Sodium Nitroprusside

**Figure 3:**
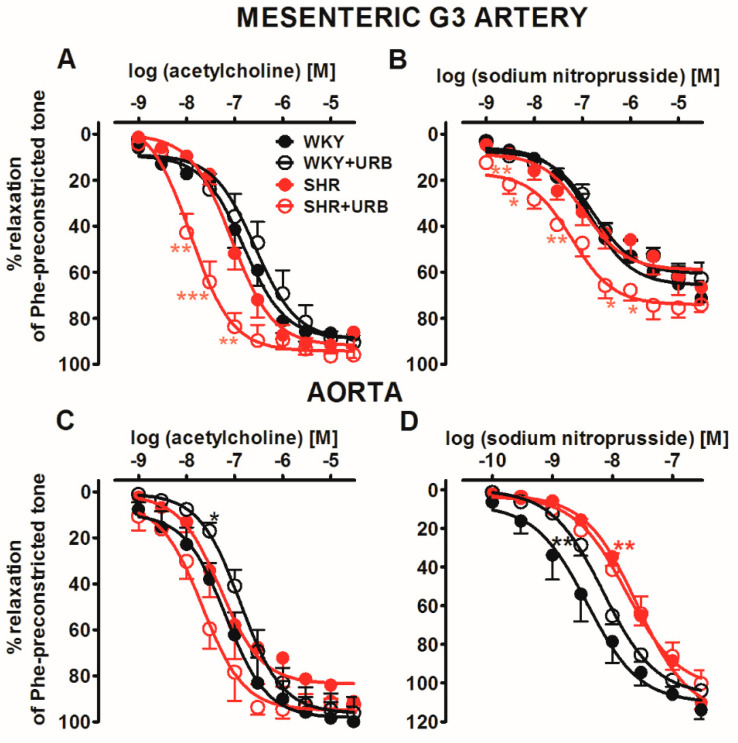
Vasorelaxant effects of acetylcholine (**A**,**C**) and sodium nitroprusside (**B**,**D**) in the mesenteric G3 arteries (**A**,**B**) and aorta (**C**,**D**) from normotensive control (WKY) and URB597-treated (WKY + URB) rats or hypertensive (SHR) and URB597-treated (SHR + URB) rats. URB597 at 1 mg/kg or its vehicle was injected intraperitoneally every 12 h for 14 days. Vasodilatory responses are shown as percentages of the reference response of the isometric contraction induced by phenylephrine (Phe). Mean ± SEM of *n* = 6 tissues for each curve. * *p* < 0.05, ** *p* < 0.01 and *** *p* < 0.001 compared to the * WKY, as determined by one-way ANOVA followed by Bonferroni’s multiple comparison tests. In a few cases, the SEM is smaller than or equal to the size of the symbols. See Table 1 and Table 2 for statistical analysis.

Next, we examined responses to Ach (mesenteric G3 arteries and aortas 0.001–30 µM) or SNP (mesenteric G3 arteries and aortas: 0.0001–30 µM) in phenylephrine-preconstricted endothelium-intact rings that cause vascular relaxation-dependent on the endothelium or smooth muscle, respectively. It allowed us to determine if the endothelium or muscle function had been changed in hypertensive animals and/or in URB597-treated rats. In normotensive rats, acetylcholine (Ach; Figure 3A,C) and sodium nitroprusside (SNP; Figure 3B,D) produced almost full (SNP in G3) or full (all other responses) concentration-dependent relaxation of isolated rat mesenteric G3 arteries (Figure 3A,B) and aortas (Figure 3C,D) preconstricted with phenylephrine. The CRCs for Ach and SNP in mesenteric G3 arteries were not changed in SHR than those for normotensive controls, with one exception: SNP-mediated vasorelaxation was shifted to the right by a factor of 6 in aortas. URB597-modified vasorelaxant responses in an artery- and blood–pressure-dependent manner. In SHR, URB597 enhanced the potency of endothelium-dependent vasorelaxation in response to Ach in mesenteric G3 arteries and aortas by 8- and 2.5-fold, respectively. Interestingly, it diminished the relaxant potency of Ach and SNP in aortas isolated from WKY by about 2-fold (for the pEC_50_ and R_max_ values, see Table 1 and Table 2; Figure 3).

### 2.4. Influence of Hypertension; an Antagonist of the CB_1_ Receptor, AM251; and Chronic Administration of URB597 on Vasodilatory Effects of Methanandamide

**Figure 4:**
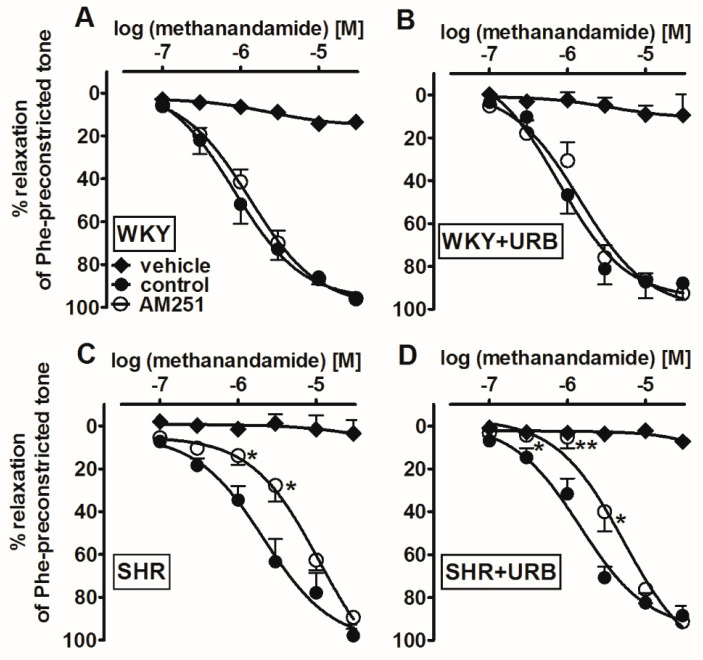
Influence of AM251 (1 µM) and its vehicle on the relaxant effects of methanandamide in the mesenteric G3 arteries from normotensive control (WKY; **A**) and URB597-treated (WKY + URB; **B**) rats or hypertensive (SHR; **C**) and URB597-treated (SHR + URB; **D**) rats. URB597 at 1 mg/kg or its vehicle was injected intraperitoneally every 12 h for 14 days. The results are expressed as percentages of the relaxation of the isometric contraction induced by phenylephrine (Phe). Mean ± SEM of *n* = 8–10 tissues for each curve. * *p* < 0.05 and ** *p* < 0.01, compared to the * control, as determined by Student’s *t*-tests for unpaired data. In a few cases, the SEM is smaller than or equal to the size of the symbols. See Table 1 for statistical analysis**.**

To check whether changes in the endocannabinoid tone elicited by hypertension and/or URB597 modified the vasorelaxant responses of cannabinoid agonist in mesenteric G3 arteries preconstricted submaximally with phenylephrine, we examined its stable analog MethAEA alone or in the presence of AM251. The stable cannabinoid receptor agonist MethAEA (0.1–30 µM), but not its vehicle, produced a concentration-dependent, almost full relaxation of endothelium-intact phenylephrine-preconstricted mesenteric G3 arteries (Figure 4A–D). MethAEA was less potent in SHR than in WKY. The maximal relaxation was comparable in all the experimental groups (for the pEC_50_ and R_max_ values, see Table 1).

Interestingly, in endothelium-intact mesenteric G3 arteries of SHR (but not WKY), the relaxation response to MethAEA was CB_1_-dependent, as the blockade of CB_1_ receptors with AM251 (1 µM) resulted in a significant ~5-fold rightward displacement of the relaxation response and reduction of its potency, but not its maximal effects (for the pEC_50_ and R_max_ values, see Table 1).

URB597 did not alter the MethAEA-induced relaxation in the mesenteric G3 arteries isolated from normotensive, as well as hypertensive, animals (Figure 4B,D). However, in the presence of AM251, the potency of MethAEA-mediated CB_1_-independent vasorelaxation in URB597-treated SHR was enhanced by about 2-fold, compared to SHR (for the pEC_50_ and R_max_ values, see Table 1 and Figure 4C,D). Notably, these observations did not apply to the WKY animals.

### 2.5. Influence of Hypertension and Chronic Administration of URB597 on Vascular Remodeling and Immunohistochemical Staining of CB_1_ Receptors

**Figure 5:**
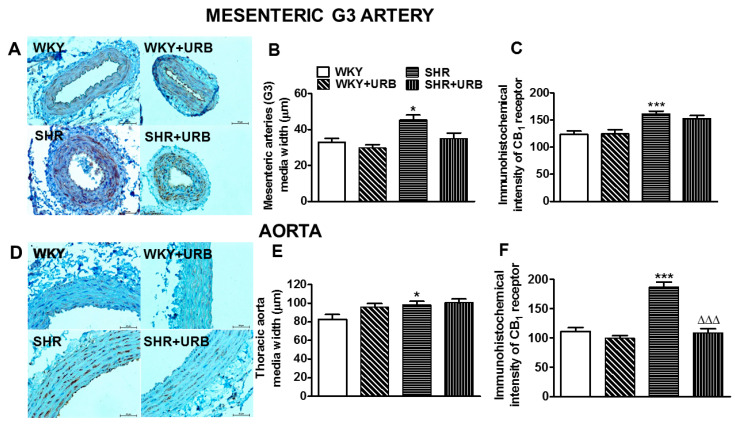
Representative micrographs of the vascular remodeling and immunohistochemical staining of CB_1_ receptors in cross-sections in the mesenteric G3 arteries (**A**) and aortic wall (**D**) from normotensive rats—control (WKY) and URB597-treated (SHR + URB). H + E staining. The bar graph illustrates the measured mesenteric G3 arteries (**B**,**C**) and aortic (**E**,**F**) medial widths and the intensity of the immunohistochemical reactions for the CB_1_ receptors, respectively. URB597 at 1 mg/kg or its vehicle was injected intraperitoneally every 12 h for 14 days. Mean ± SEM of *n* = 5 tissues for each bar; * *p* < 0.05 and ***^,∆∆∆^ *p* < 0.001 compared to the respective control groups (* WKY or ^∆^ SHR); bar = 50 μm.

Representative images of the vascular remodeling and immunohistochemical staining of CB_1_ receptors in cross-sections of mesenteric G3 arteries and aortas are shown in Figure 5. Hypertension induced medial hypertrophy in mesenteric G3 arteries and aortas by approximately 40% and 20%, respectively. The immunostaining of the CB_1_ receptor revealed its presence in the endothelial and smooth muscle cells of the walls of the mesenteric G3 arteries and aortas in all the groups. More intense staining of CB_1_ receptors, by about 30% and 70%, was observed in the endothelium and smooth muscle cells of mesenteric G3 arteries and aortas from SHR than in those from WKY respectively (Figure 5A,C,D,F).

URB597 tended to decrease the mesenteric G3 artery wall thickness in SHR to the control level (Figure 4A,B), but it did not modify the hypertrophy of the aortas (Figure 5D,E). It reduced CB_1_ receptor immunostaining to the level obtained in WKY in aortas only (Figure 5D,F).

### 2.6. Influence of Hypertension and Chronic Administration of URB597 on Expression of CB_1_ and FAAH in Isolated Mesenteric G3 Arteries and Aorta

**Figure 6:**
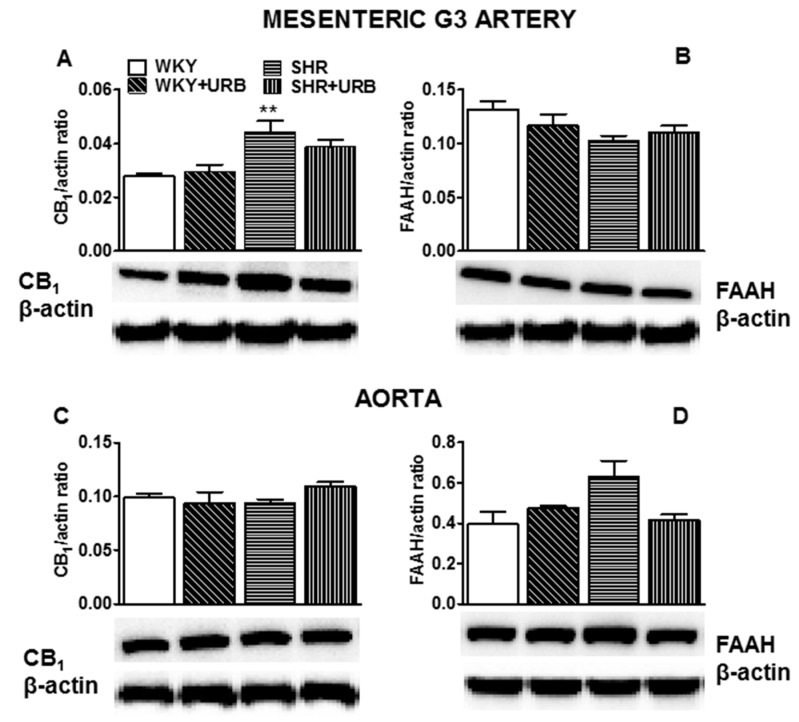
Western blots of CB_1_ (**A**,**C**) and fatty acid amide hydrolase (FAAH; **B**,**D**) protein in the mesenteric G3 arteries (**A**,**B**) and aortas (**C**,**D**) of normotensive rats—control (WKY) and URB597-treated (WKY + URB)—or hypertensive rats—SHR and URB597-treated (SHR + URB). URB597 at 1 mg/kg or its vehicle was injected intraperitoneally every 12 h for 14 days. On the bottom of the panel, representative Western blots for CB_1_, FAAH, and β-actin (which served as the loading control) are shown. Mean ± SEM of 5–6 rats; ** *p* < 0.01 compared to the control group (* WKY).

The expression of CB_1_ receptors and FAAH in isolated mesenteric G3 arteries and aortas were analyzed by Western blotting. It showed a single immunoreactive band of the molecular size expected for CB_1_ receptors (60 kDa) and FAAH (67 kDa) in both vessels (*n* = 5–6). The expression of CB_1_ receptors, but not FAAH, increased in mesenteric G3 arteries from SHR (Figure 6A,B). We did not observe any significant changes in the expression of cannabinoid-related proteins in isolated aortas subjected to various experimental conditions (Figure 6C,D).

Western blot analysis also revealed that URB597 failed to modify the expression of the examined cannabinoid-related proteins (Figure 6A–D).

### 2.7. Influence of Hypertension and Chronic Administration of URB597 on Endocannabinoid Levels in Isolated Mesenteric G3 Arteries and Aortas

**Figure 7:**
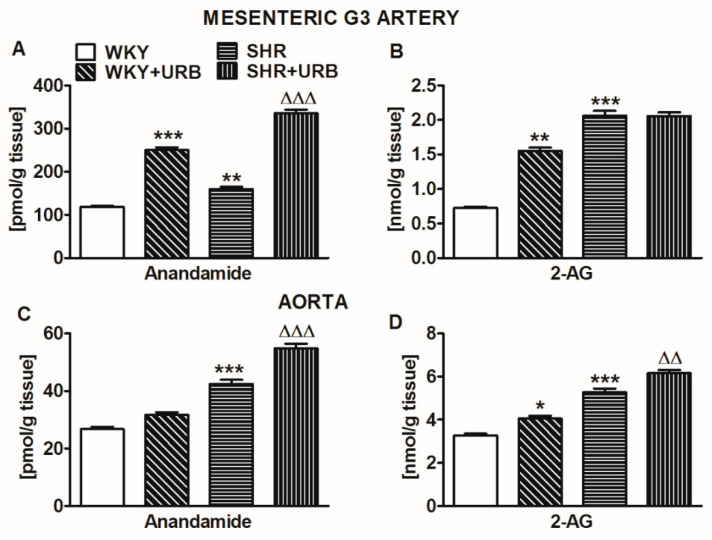
Concentrations of anandamide (**A**,**C**) and 2-arachidonoylglycerol (2-AG; **B**,**D**) in the mesenteric G3 arteries (**A**,**B**) and aortas (**C**,**D**) of normotensive rats—control (WKY) and URB597-treated (WKY + URB)—or hypertensive rats—SHR and URB597-treated (SHR + URB). URB597 at 1 mg/kg or its vehicle was injected intraperitoneally every 12 h for 14 days. Data are presented as mean ± SEM (*n* = 6 per group); * *p* < 0.05, **^,∆∆^ *p* < 0.01, and ***^,∆∆∆^ *p* < 0.001 compared to the respective control groups (* WKY or ^∆^ SHR).

As shown in Figure 7, the anandamide level in small mesenteric G3 arteries was approximately 4 times higher than that in aortas from WKY. By contrast, the 2-arachidonoyl glycerol (2-AG) level was approximately 4 times lower in G3 than in aortas. The 2-AG level in normotensive WKY was approximately 6 and 120 times higher than the respective values of anandamide in mesenteric G3 arteries and aortas. In SHR, the anandamide and 2-AG levels were about 35% and 180% higher, respectively, in mesenteric G3 arteries and about 60% higher in aortas (both endocannabinoids).

URB597 markedly enhanced the vascular levels of anandamide (by about 100% in mesenteric G3 arteries and 30% in aortas) in hypertensive rats; however, in WKY, its content was increased by about 100% only in mesenteric G3 arteries. In addition, the chronic inhibition of FAAH elevated the levels of 2-AG by about 115% and 25% in mesenteric G3 arteries and aortas of WKY, respectively, and by about 20% in the aortas of SHR (Figure 7).

## 3. Discussion

We examined whether endocannabinoids and cannabinoid CB_1_ receptors play a protective role in developing local structural and functional changes in the vasculature under a hypertensive condition and when the endocannabinoid system is pharmacologically over-activated. For this purpose, we used the most common animal model of primary hypertension, SHR, which responds to almost all classes of the approved antihypertensive drugs [22], and normotensive controls, WKY, which were chronically treated with the FAAH inhibitor URB597 (1 mg/kg/12 h for 2 weeks). Such dosing almost completely (~90%) inhibited the cardiac FAAH activity in hypertensive animals 12 h after the final dose and, consequently, increased cardiac and plasma anandamide in SHR and DOCA-salt [23], as well as reduced blood pressure in DOCA-salt [11,20]. Therefore, it is reasonable to expect the vascular FAAH inhibition similar to that observed previously in the rat heart. We examined two different types of isolated endothelium-intact vessels: resistance (mesenteric G3 arteries) and conduit (aortas) because (1) vascular changes related to hypertension and cannabinoids vary, depending on the vessel size (for literature, see the Introduction), and (2) FAAH activity strongly depends on functional endothelium. Thus, URB597 enhanced anandamide (but not its stable analog MethAEA)-induced relaxation only in the endothelium-intact, but not in the denuded, isolated rat small mesenteric artery [24,25]. Moreover, endothelial denudation reduced the amplificatory influence of URB597 on the relaxation elicited by anandamide in rat mesenteric G3 arteries [25] and completely inhibited the anandamide-induced relaxation of the rat aortas [26]. We used the stable anandamide analog MethAEA (K_i_ values, 17.9–28.3; [27]) as a CB_1_ receptor agonist, which has shown a relaxant ability in small and large arteries [4,28] and which allowed us to avoid the vascular effects of anandamide-related metabolites.

### 3.1. Vascular Changes Related to Hypertension

In our study, we confirmed the typical vascular changes related to hypertension (reviewed, for example, by [3,22,29]). Thus, in both mesenteric G3 arteries and/or aortas, we determined significant wall hypertrophy that led to a reduced lumen diameter and a thickening of the vascular media, connected with the enhanced vasoconstrictive responses to U46619 and/or phenylephrine. In contrast to the above changes, endothelium-dependent relaxation in response to Ach was unchanged in small mesenteric G3 arteries and in aortas, while the endothelium-independent vasorelaxant effect of SNP was reduced in aortas only. Similarly, impairment of endothelium-independent vasorelaxation has been observed as characteristic vascular target-organ damage in conduit arteries [22]. On the other hand, the endothelial function in SHR may be impaired, unaltered, or improved, depending on age, artery type, and the methods used to determine vascular function [30]. In clinical studies in patients with mild essential hypertension, small vessel remodeling has been the most prevalent, whereas only 60% had endothelial dysfunction [22,31].

This is the first study demonstrating that anandamide and 2-AG levels increased in both resistance and conduit vessels of SHR, compared with the levels in normotensive controls, without any changes in FAAH expression. Interestingly, the plasma and cardiac levels of anandamide and 2-AG decreased in SHR but increased in DOCA-salt [23,32] and were not changed in the lungs of a rat experimental pulmonary hypertension model [33]. Moreover, they tend to be higher in aortic [34] or cardiac [23,32] tissue than in peripheral plasma. These differences indicate that the levels of endocannabinoids depend on the tissue and model of hypertension. In our study, the concentration of anandamide was about 5 times higher in small mesenteric G3 arteries than in the aorta. On the contrary, the 2-AG content was higher in aortic tissue than in small resistance vessels. Similar decreases in 2-AG concentration were noticed in humans, from the aortic root to the peripheral arteries [34]. The concentrations of anandamide were approximately 6- to 100-fold lower than the respective 2-AG levels in resistance and conduit arteries, both normotension and hypertension. Importantly, one should bear in mind that higher 2-AG levels in the aortic tissue have been demonstrated to promote atherogenesis in mice [35].

Our results demonstrate that cannabinoid CB_1_ receptors activated by endocannabinoids may play a local role in protecting against hypertension development. First, the CB_1_ receptor antagonist AM251 (1 μM) enhanced the vasoconstrictive responses to U46619 (potency and efficacy) and phenylephrine (potency) in small mesenteric G3 arteries, but not in aortas isolated from both SHR and WKY. These results revealed that the contractions induced by the above agents were diminished by the CB_1_-dependent vasodilatory effects of endocannabinoids. They are following the previous suggestion that the production of endocannabinoids in the vasculature and, therefore, the degree of inhibition of vessel contraction may be dependent on agonist-induced contraction force ([19]; in our hands, U46619 is a more potent vasoconstrictor than phenylephrine). This novel CB_1_-dependent negative feedback mechanism, which restrains the increase in vascular tone, has mainly been described under in vitro conditions (for literature, see the Introduction, Figure 1). By contrast, its potential significance in vivo in hypertension has, to date, been suggested based only on the observation that the increase in blood pressure induced by a 10 min infusion of angiotensin II was higher in CB_1_ receptor knockout mice than in their respective controls [15].

Second, the expression of CB_1_ receptors clearly increased in the vasculature (both mesenteric G3 and aortas) of SHR, as confirmed by Western blotting and/or immunohistochemistry. The upregulation of CB_1_ receptors in hypertension has been demonstrated previously in rat small mesenteric G3 arteries and in aortas (for literature, see the Introduction).

Third, the changes in CB_1_ receptor density in the current study correlated with the results of functional experiments, in which the reduction of vasorelaxation in response to MethAEA in mesenteric G3 arteries was sensitive to AM251 in SHR, but not in WKY, indicating that it was mediated by CB_1_ receptors in SHR, but not in WKY. It seems that the vasodilatory effects of cannabinoids depend on the model of hypertension. Thus, similar attenuations of responses to MethAEA or anandamide have been observed in perfused and isolated mesenteric arteries from SHR [7,24] and CBD [5], while the enhancement of the MethAEA- and CBD-induced relaxation has been observed in small mesenteric G3 arteries of DOCA-salt rats [4,5], respectively, and aortas isolated from renal hypertensive rats [13].

In summary, our results suggest that vasodilatory endocannabinoid levels are enhanced in SHR, acting through the activation of upregulated CB_1_ receptors in resistance arteries, which may diminish the increased contractions elicited by various factors. Furthermore, it is a form of local protective feedback, as we determined it in mesenteric G3 arteries, but not in aortas.

### 3.2. Vascular Changes Induced by URB597 in SHR and in WKY

The effectiveness of chronic URB597 (1 mg/kg twice daily for two weeks) administration in hypertensive and normotensive rats was confirmed by increases in anandamide levels in both types of vessels isolated 12 h after the last dose. The only exception was no significant changes in the anandamide level in the aortas of WKY. Moreover, in aortas of SHR and in both types of vessels isolated from WKY, we observed increases in 2-AG levels, resulting from the fact that URB597 also partially inhibits the enzyme responsible for the degradation of this endocannabinoid (see, for example, [23]).

We demonstrated that chronic URB597 administration is locally beneficial in hypertensive rats, as it modifies, in a vessel size-dependent fashion, the function and morphology of the vasculature. Thus, in small mesenteric G3 arteries, (1) it improved endothelium-dependent vasorelaxation occurring in response to Ach; (2) in the presence of CB_1_ receptor blockade, it reduced the vasoconstrictive response to phenylephrine and enhanced the vasodilatory effect of MethAEA; and (3) it reversed the hypertrophy of mesenteric G3 arteries to the level of that in control rats. In aortas, URB597 (1) enhanced the endothelium-dependent vasorelaxant effect of Ach and (2) decreased CB_1_ protein expression.

How can we explain the above changes? Anandamide and its stable analog MethAEA relax blood vessels not only by the activation of CB_1_ receptors but also by transient receptor potential vanilloid type 1 (TRPV1), sensitively to O-1918, cannabinoid CB_2_ receptors, and/or K_Ca_ (i.e., for aortas [13,28] and small mesenteric G3 arteries [4,7,36], Figure 1). In our study, URB597 increased anandamide levels, which enhanced the vasorelaxation induced by Ach (in mesenteric G3 arteries and aortas, mediated by CB_1_ receptors and other above-mentioned non-CB_1_ targets, described in details in Figure 1) and, independently of CB_1_ receptors, diminished phenylephrine-evoked contraction and increased the relaxation stimulated by MethAEA in mesenteric G3 arteries. Importantly, all of the above effects of URB597 were noticed in SHR but not in their normotensive controls. Similarly, the enhancement of the vasodilatory effect of Ach in the presence of increased endogenous or exogenous (i.e., cannabidiol) cannabinoids has previously been demonstrated in diabetic and hypertensive rats, but not in lean or normotensive control animals [1,37,38]: that is, only in the case of increased endocannabinoid levels and vascular dysfunction (for a review, see [1,2,39]). This is consistent with the known pro-homeostatic properties of the endocannabinoid system, which plays a crucial role in a perturbed system, but not in a healthy one.

The antihypertensive activities of drugs might also result from their indirect effects on the vascular wall. The degree of remodeling of the media layer and the underlying mechanisms of hypertension depends on the vessel size [40,41]. Thus, the hypertrophy of conductance arteries is pressure-dependent, while hyperplastic changes in small arteries are mainly tone-dependent [40,41]. Accordingly, the correction of the structure of small mesenteric arteries in URB597-treated SHR might be a consequence of the vasodilation improvement, thus lowering peripheral resistance, while the lack of correction of the aorta wall morphology may—at least partially—result from unchanged systemic blood pressure. Similarly, chronic URB597 administration has been previously demonstrated to prevent the elevation of the wall thickness of intrapulmonary arteries in mice [42] and reduced aortic hypertrophy in DOCA-salt rats [4], connected with a decrease in hypoxia-induced pulmonary hypertension and drop in blood pressure, respectively.

Chronic URB597 administration failed to diminish blood pressure in SHR ([20], and the current study). CB_1_ receptors are partially responsible for the relaxation of the aorta (also in hypertension; see [4,13]). Thus, the decrease in their density in aortas in response to the FAAH inhibitor and lack of changes in the hypertension-induced hypertrophy in this vascular bed may be—at least, to some extent—responsible for the lack of hypotensive influence of URB597. The antihypertensive potential of the endocannabinoid-degrading enzyme FAAH has been demonstrated to depend on the hypertension model, with more pronounced changes in, for example, the DOCA-salt model [1,20], including vascular changes that were at least partially responsible for the drop in blood pressure [4].

Chronic URB597 increased vascular anandamide levels but did not modify the local protective feedback we observed in mesenteric G3 arteries isolated from hyper- and normotensive rats, which led to a decrease in vasoconstriction elicited by phenylephrine and U46619 by endocannabinoids acting through CB_1_ receptors. However, it has been previously determined that this mechanism is not sensitive to FAAH but is enhanced only by MAGL inhibitors in human and rat pulmonary arteries [18] or in rat middle cerebral arteries [17], demonstrating the involvement of 2-AG, but not AEA in this effect (as reviewed by [19]). In our study, URB597 increased 2-AG content in aortas only, in which this negative feedback was not present. Surprisingly, URB597 enhanced (rather than diminishing) the U46619-induced vasoconstriction of mesenteric G3 arteries. We can only speculate that this resulted from the conversion of 2-AG to the constrictor prostanoid thromboxane TXA_2_ [43].

URB597, in the context of vascular effects, seems to be a safe drug. The only effect of its chronic administration was the attenuation of endothelium-dependent and independent relaxation in aortas of WKY, possibly due to a lack of increase in anandamide content. By contrast, URB597 enhanced the anandamide and 2-AG contents in mesenteric G3 arteries but without any direct relaxant effect. However, in our previous studies, we observed the unexpected and unwanted effects of chronic URB597 administration in normotensive controls, including impaired Ach-induced vasodilatation and potentiated phenylephrine-induced vasoconstriction in small resistance G3 arteries [4], increased cardiac diastolic stiffness, and modified cardiostimulatory effects of isoprenaline [20], among others. Thus, caution should be taken when studying cannabinoids and FAAH inhibitors as potential therapeutics due to their vessel- and model-specific activities, and the side effects connected with off-target responses and the activation of alternative pathways of anandamide metabolism.

### 3.3. Limitations

We determined that chronic URB597 administration to rats with primary (SHR, the current study) and secondary (DOCA-salt, [4]) hypertension-induced cardiovascular changes dependent on the model of hypertension. In this context, it would be interesting to examine its influence in rats with renin–angiotensin–aldosterone system-dependent hypertension, in which a cannabinoid CB_1_ receptor antagonist has been shown to decrease blood pressure, in contrast to the increase in SHR (see [1]). Moreover, the basal SBP before the first dose of URB597 was almost 30% higher in DOCA-salt [4] than in SHR (the current study), which was connected with a respectively higher and lower degree of endothelium dysfunction and various genetic and environmental triggers or key pathophysiological mechanisms characterizing both models of hypertension [5,22]. Thus, we cannot exclude a more pronounced beneficial effect of URB597 in older SHR with more pronounced endothelium dysfunction and/or higher blood pressure. In addition, sexual dimorphism was observed in the vasodilatory effect of AEA in mesenteric arteries isolated from WKY and SHR animals [24]. The question arises whether URB597 would be an effective hypotensive if we used female SHR instead of males, as, in SHR, the response to anandamide in mesenteric G3 arteries was decreased in hypertensive males but not changed in female rats. We determined that the CB_1_ receptor-dependent vascular feedback protecting against vasoconstrictor compounds involves 2-AG rather than anandamide [18,19]. As such, it would be interesting to examine the vascular effects of the chronic administration of a MAGL inhibitor or dual FAAH/MAGL inhibitor in hypertension. Nevertheless, although URB597 (also known as KDS4103) is the most investigated FAAH inhibitor, it is not exclusively selective for FAAH [2]. Moreover, MethAEA also activates other receptors [4,7,13,28,36]. Consequently, it is necessary to consider other possible molecular targets for FAAH- and MethAEA-mediated signaling pathways in hypertension.

## 4. Material and Methods

### 4.1. Animals

Male 10–12 week-old SHR and Wistar-Kyoto (WKY) rats that weighed 280–310 g were purchased from the Center for Experimental Medicine of the Medical University of Białystok. All animal care, surgical procedures and experimental protocols were performed following the European Directive (2010/63/EU) and Polish legislation and were approved by the local Animal Ethics Committee in Olsztyn (Poland, project code: 81/2017, approved 28 November 2017). Animal studies are reported in compliance with the ARRIVE guidelines [44]. The study was performed in compliance with the principles of replacement, refinement or reduction (the 3Rs). Animals were housed at constant humidity (60 ± 5%) and temperature (22 ± 1 °C) and were kept under a 12/12 h light/dark cycle. They were maintained on standard pelleted rat chow and tap water ad libitum unless otherwise noted.

Hypertensive and normotensive rats were injected intraperitoneally (i.p.) with URB597 (1 mg/mL/kg, i.e., ~3 μmol/kg) for 14 days every 12 h. Control animals in each group received a vehicle for URB597 (1 mL/kg; DMSO, Tween-80 and 0.9% NaCl (1:2:7)) [20]. Two experimental groups were created in normotensive rats: (I) control—WKY, (II) URB597 treated—WKY + URB; and two in SHR: (III) SHR and (IV) URB597-treated—SHR + URB.

Systolic blood pressure was measured in conscious rats by a non-invasive tail-cuff method (using a rat tail blood pressure monitor (Hugo Sachs Elektronik-Harvard Apparatus, March–Hugstetten, Germany)). Animals with SBP equal to or higher than 150 mmHg were considered hypertensive and underwent a myography procedure and biochemical and histochemical evaluations.

### 4.2. Vessel Preparation

Twelve hours after the last dose of URB597 or its vehicle, rats were anesthetized with pentobarbitone sodium (70 mg/kg, i.e., 300 µmol/kg i.p.). The vessel preparation and experimental procedure have been described in detail previously [4,5]. Following sacrifice, the aorta and mesenteric arterial bed were removed rapidly and placed into a cold Krebs-Henseleit solution with the following composition (in mM) NaCl 118; KCl 4,8; CaCl_2_ 2.5; MgSO_4_ 1.2; NaHCO_3_ 24; KH_2_PO_4_ 1.2; glucose 11; and EDTA 0.03 at pH 7.4. From the mesenteric arterial bed, 2 mm segments of the third-order of the superior artery (G3) were dissected free of adherent connective and adipose tissue. Arterial segments [~250 µm internal diameter] were then mounted in a Mulvany–Halpern-type wire myograph (Model 620 M, Danish Myo Technology, Aarhus, Denmark). Tension was measured and was recorded on the LabChart 7.3.7 Pro (ADInstruments, Hastings, UK). The thoracic aortas (3–5 mm long) were cleaned of adherent tissue and suspended on stainless-steel wires in 10-mL organ baths. Muscle tension was recorded by a force-displacement transducer (PIM 100RE, BIO-SYS-TECH, Białystok, Poland) and displayed on a computer. All vessels were kept at 37 °C in gas with 95% O_2_ and 5% CO_2_ Krebs-Henseleit solution and were allowed to equilibrate for 45 min (resting tension ~2.5 mN) for mesenteric G3 arteries and for 60 min (resting tension ~14.7 mN) for thoracic aortas.

### 4.3. Concentration–Response Curves

After a stabilization period, each vessel was initially precontracted with high 120 mM KCl followed by washout. Then, the integrity of the endothelium was assessed by preconstricting rings submaximally with the α_1_-adrenoceptor agonist (–)-phenylephrine (mesenteric G3 arteries: 3–10 μM and aorta: 0.3 µM rats), followed by relaxations with Ach 10 or 1 µM, respectively. We considered a relaxation response of at least 90% to Ach to be an endothelium-intact vessel. After washout, CRCs were constructed by the cumulative addition of appropriate agonists (in each preparation, only one experimental curve was determined).

To test the vascular contractile function, endothelium-intact mesenteric G3 arteries and aortas were exposed to phenylephrine: 0.01–30 µM and 0.001–30 µM and thromboxane analog U46619 0.001–3 µM and 0.0001–0.3 µM, respectively. To determine if the endothelium or muscle function was changed in hypertensive animals and/or URB597-treated rats, we exposed phenylephrine-preconstricted endothelium-intact rings from each group (I–IV) to Ach (mesenteric G3 arteries and aortas 0.001–30 µM) or SNP (mesenteric G3 arteries and aortas: 0.0001–30 µM). The vasorelaxant responses of cannabinoid agonist, MethAEA (0.1–30 µM) was tested on mesenteric G3 arteries that were preconstricted submaximally with phenylephrine. Appropriate vehicle control was obtained by adding Tocrisolve (MethAEA) to the preconstricted arteries. All experiments were performed in paired vessels—the vehicle control responses were compared with the drug-treated group response on vessels from the same rat.

To verify the involvement of CB_1_ receptors in the vasoconstriction to phenylephrine or U466119 and to vasorelaxant effects of MethAEA in mesenteric G3 arteries and/or aortas, rings were pretreated with the CB_1_ receptor antagonist: AM251 for 45 min (1 µM, [5]), that was present throughout the remainder of the experiment. In control tissues, the respective vehicle was used instead. At the end of the CRCs, each vessel was again contracted with high 120 mM KCl to determine the vessel viability.

### 4.4. Western Blots

Routine Western blotting procedures were used as described previously [4]. Briefly, samples of mesenteric G3 arteries and aortas were harvested and then lysed and homogenized in a radioimmunoprecipitation assay (RIPA) buffer containing a cocktail of protease inhibitors (Roche Diagnostics GmbH, Mannheim, Germany). The total protein concentration was determined using the bicinchoninic acid method with bovine serum albumin as a standard. Next, homogenates were reconstituted in Lemmli buffer, separated by 10% sodium dodecyl sulfate-polyacrylamide gel electrophoresis and transferred onto nitrocellulose membranes. The membranes were incubated overnight at 4°C with corresponding primary antibodies in appropriate dilutions (i.e., CB_1_ (1:500; Abcam, Cambridge, UK), FAAH (1:200; Santa Cruz Biotechnology, Santa Cruz, CA, USA) and actin (1:1000; Sigma-Aldrich, Saint Louis, MO, USA). Thereafter, to detect proteins, anti-rabbit and anti-goat IgG horseradish peroxidase-conjugate secondary antibodies (1:3000; Santa Cruz Biotechnology, Santa Cruz, CA, USA) were used. Equal protein concentration-loading was controlled by Ponceau S staining. After adding a suitable substrate for horseradish peroxidase (Clarity Western ECL substrate; Bio-Rad, Hercules, CA, USA), the protein bands were quantified densitometrically using a ChemiDoc visualization system EQ (Bio-Rad, Warsaw, Poland). The levels of the protein detected were normalized to actin.

### 4.5. Thickness of Media in Mesenteric G3 Arteries and Aortas

Mesenteric G3 arteries and thoracic aortas were fixed in 10% buffered formalin and routinely embedded in paraffin. Sections were cut to be 4 μm in thickness (by a Leica 2025 rotating microtome) and stained by hematoxylin and eosin (H + E). The quantitative analysis of the preparations and their photographic documentation was performed with an Olympus BX41 light microscope and a video circuit and Pentium 120 PC with NIS Elements BR software for microscope image analysis. The measurement of the thickness of the middle layer of the examined blood vessel wall was made at a uniform magnification of 200 (×10 objective and ×20 eyepiece). Five sections from each artery were measured.

### 4.6. Immunohistochemistry

In the immunohistochemical study, the EnVision method was used according to Baranowska-Kuczko et al. [5] using an antibody against the cannabinoid CB_1_ receptor [1:1000; rabbit Anti-CB_1_ (no cat. ab23703); Abcam, Cambridge, MA, UK]. The specificity of the antibody included a negative control, in which the antibodies were replaced by normal rabbit serum (Vector Laboratories, Burlingame, CA, USA) at the respective dilution (no staining), and positive control, where the tissue recommended by the antibody manufacturer was used for staining, which was prepared: for CB_1_ from human cerebellum. All control reactions performed gave positive results. The obtained results of immunohistochemical staining were evaluated on the Olympus BX41 microscope with the Olympus DP12 camera under a magnification of 200× (20× lens and 10× eyepiece; each field was 0.785 mm^2^) and submitted to morphometric evaluation by using software for image analysis Nikon Elements Advanced Research software (NIS). In each blood vessel, the intensity of the immunohistochemical reaction was measured in ten randomly selected sites using a 0 to 256 grayscale level in which completely black pixels were given a value of 256, and white or bright pixels were given a value of 0.

### 4.7. Determination of Endocannabinoids

Mesenteric G3 arteries and aortas were snap-frozen and held at −80 °C and pulverized in liquid nitrogen to examine levels of endocannabinoids. Anandamide (AEA) and 2-arachidonoylglycerol (2-AG) were quantified using modified ultrahigh performance liquid chromatography-tandem mass spectrometry LC–MS (LC–MS 8060, Shimadzu, Kyoto, Japan) by Luque-Córdoba method [45]. Octadeuterated endocannabinoids: AEA-d8 and 2-AG-d8 as internal standards were added into the samples, and all endocannabinoids were isolated using a solid phase extraction (OASIS HLB 3cc). Endocannabinoids were separated on an Agilent Poroshell 120 EC-C18 analytical column (3.0 × 150 mm, 2.7 µm particle size; Agilent, Santa Clara, CA, USA). The mobile phase consisted of 0.1% formic acid in water (A) and 0.1% formic acid in acetonitrile (B). The gradient conditions were as follows: 70% B to 80% B in 5.0 min, 80% B to 88% B in 15.0 min, 88% B to 100% B in 16.0 min, held at 100% B from 16.0 to 20.0 min, 100% B to 70% B at 21.0 min and maintained at 70% B to re-equilibrate the column until the end of runtime (4 min). The samples were analyzed in positive-ion mode using multiple reaction monitoring (MRM). Transitions of the precursor to the product ion were as follows: *m/z* 348.3→62.1, *m/z* 379.3→287.2, *m/z* 356.3→63.1, *m/z* 387.0→ 295.0 (for AEA, 2-AG, AEA-d8 and 2-AGd8, respectively).

### 4.8. Drugs

N,N-dimethylformamide (DMF) and Tween-80 (Sigma-Aldrich, Steinheim, Germany); URB597 [(3’-(aminocarbonyl)[1,1’-biphenyl]-3-yl)-cyclohexylcarbamate, anandamide-d8 (AEA-d8), 2-arachidonoyl glycerol-d8 (2-AG-d8), (Cayman Chemical Company, Ann Arbor, MI, USA); pentobarbitone sodium (Biowet, Puławy, Poland) and sodium chloride (NaCl) (Chempur, Piekary Śląskie, Poland).

Acetylcholine chloride, phenylephrine, and sodium nitroprusside (Sigma, Munich, Germany) were dissolved in deionized water. Methanandamide (Tocris, Bristol, UK) was purchased as a 10 mg/mL emulsion in soya water (1:4) and was further diluted with deionized water. Stock solution (10 µM) of AM251 (N-(piperidin-1-yl)-5-(4-iodophenyl)-1-(2,4-dichlorophenyl)-4-methyl-1H-pyrazole-3-carboxamide) and U46619 (9,11-methanoepoxy PGH_2_) (Tocris, Bristol, UK) were prepared in ethanol (0.1% *v/v*). The final concentrations of these agents were prepared by dilutions with deionized water, which adjusted the final concentrations of ethanol to less than 0.1% *v/v* and 0.01% *v/v*, respectively. The antibodies used in the Western blots were purchased from Abcam (Cambridge, UK; anti-CB_1_; cat. no. ab23703), Santa Cruz Biotechnology (Santa Cruz, CA, USA; anti-FAAH, cat no sc-26427; anti-rabbit IgG horseradish peroxidase-conjugate secondary antibody, sc-2004; anti-goat IgG horseradish peroxidase-conjugated secondary antibody, cat. no. sc-2020) and Sigma-Aldrich (Saint Louis, MO, USA; anti-actin, cat. no A2668). Reagents for routine histological H + E staining and secondary antibody EnVision + kit HRP rabbit were obtained from Dako Denmark A/S (Glostrup, Denmark), and normal rabbit serum was from Vector Laboratories (Burlingame, CA, USA).

### 4.9. Statistical Analysis

For contractile responses to phenylephrine and U46619, data are shown as a percentage of the reference response to the 120 mM KCl given after equilibration at the beginning of the experiments. All of the relaxant effects produced by Ach, SNP and MethAEA were expressed as the percentage relaxation of the tone induced by phenylephrine. GraphPad Prism 5.0 software (CA, USA) was used to plot the mean data as sigmoidal concentration–response curves. The curves were used to determine the potency (pEC_50_ the negative logarithm of the concentration causing the half-maximum effect) and maximal response (R_max_) values. In analogy to our previous study [5], if the CRC did not reach a clear top plateau, it was quantified as a rough measure of the maximum extent of relaxation obtainable (R_max_). The rightward shifts of CRCs relative to the control curve were calculated based on the EC_50_ values. All of the results are expressed as the mean ± SEM of *n* animals. Intergroup statistical comparisons were made by analysis of variance (ANOVA) followed by Bonferroni’s multiple comparison test of the entire data set. Post hoc tests were run only if F achieves the necessary level of statistical significance and there was no significant variance in inhomogeneity. The Student’s *t*-test for unpaired data was used as appropriate. Differences were considered significant at *p* < 0.05.

## 5. Conclusions

We found that the chronic administration of URB597 (1 mg/kg twice daily for two weeks), an inhibitor of the enzyme responsible for anandamide degradation, increased vascular anandamide and 2-AG levels and caused locally beneficial changes for primary hypertension, in terms of the function and morphology of the vasculature, in a vessel-size-dependent manner. We speculate that FAAH activity and CB_1_ receptors are regionally restricted to resistance arteries. Thus, in small mesenteric G3 arteries, URB597 diminished the wall hypertrophy, enhanced the vasodilatory effect of Ach and, in the presence of CB_1_ receptor blockade, increased MethAEA-stimulated relaxation and reduced phenylephrine-induced vasoconstriction. By contrast, in aortas, it only augmented the response to Ach. Cannabinoid CB_1_ receptors located in mesenteric G3 arteries were upregulated in SHR, which has been demonstrated to play a protective role in hypertension, as they mediated the vasorelaxation induced by MethAEA (this effect was not determined in normotension). Moreover, their activation by endocannabinoids, the levels of which were enhanced in hypertension, diminished the vasoconstriction induced by various compounds. Such local vascular positive effects of FAAH inhibitors may have additional benefits during the therapeutic application of the pharmacological inhibition of FAAH (for a review, see [2]).

## Figures and Tables

**Table 1:** The influence of AM251 (1 µM) on the vasoconstriction to phenylephrine (Phe), thromboxane analog U46619 and vasorelaxation to methanandamide (MethAEA) and vasorelaxant effects of acetylcholine (Ach) and sodium nitroprusside (SNP) in the endothelium-intact isolated small mesenteric G3 arteries from normotensive rats: control (WKY) and URB597-treated (WKY + URB), or hypertensive rats: (SHR) and URB597-treated (SHR + URB).

Group	WKY	WKY + URB	SHR	SHR + URB
**Phe**	(6)	(6)	(6)	(6)
pEC_50_	5.3 ± 0.10	5.4 ± 0.10	5.6 ± 0.07 *	5.5 ± 0.10
R_max_ (%)	129.9 ± 13.4	113.0 ± 4.6	122.8 ± 6.9	116.0 ± 6.3
**Phe + AM251**	(6)	(6)	(6)	(6)
pEC_50_	5.7 ± 0.10 ^#^	5.6 ± 0.10	6.1 ± 0.07 *^,###^	5.7 ± 0.08 ^∆^
R_max_ (%)	158.4 ± 16.2	142.2 ± 12.6	144.9 ± 14.3	148.1 ± 22.3
**U46619**	(6)	(6)	(6)	(6)
pEC_50_	6.1 ± 0.05	6.2 ± 0.04	6.5 ± 0.05 ***	7.0 ± 0.07 ^∆∆∆^
R_max_ (%)	76.8 ± 9.1	72.7 ± 7.6	75.5 ± 5.6	88.9 ± 7.0
**U46619 + AM251**	(6)	(6)	(6)	(6)
pEC_50_	6.8 ± 0.03 ^&&&^	6.5 ± 0.06 ^&&^	6.9 ± 0.10 ^&&^	7.2 ± 0.09
R_max_ (%)	87.1 ± 6.3	97.0 ± 4.4^&^	90.2 ± 4.4	111.2 ± 5.7 ^&^
**Ach**	(6)	(6)	(6)	(6)
pEC_50_	6.8 ± 0.05	6.6 ± 0.09	7.0 ± 0.07	7.9 ± 0.07 ^∆∆∆^
R_max_ (%)	87.4 ± 4.4	90.6 ± 3.4	86.1 ± 11.1	96.0 ± 3.1
**SNP**	(6)	(6)	(6)	(6)
pEC_50_	6.8 ± 0.09	6.8 ± 0.10	7.0 ± 0.10	7.2 ± 0.10
R_max_ (%)	71.2 ± 7.5	62.7 ± 6.9	66.7 ± 7.3	74.2 ± 3.1
**MethAEA**	(10)	(10)	(8)	(8)
pEC_50_	6.1 ± 0.07	6.1 ± 0.10	5.6 ± 0.10 **	5.8 ± 0.10
R_max_ (%)	96.5 ± 1.7	87.8 ± 7.7	97.9 ± 3.1	88.4 ± 4.4
**MethAEA + AM251**	(10)	(10)	(8)	(8)
pEC_50_	5.9 ± 0.04	5.8 ± 0.10	4.9 ± 0.07 ***^,$$$^	5.2 ± 0.10 ^∆∆∆,$$^
R_max_ (%)	96.0 ± 1.3	92.6 ± 4.1	89.4 ± 3.2	91.3 ± 1.5

URB597 1 mg/kg or its vehicle was injected intraperitoneally every 12 h for 14 days. Data are expressed as the mean ± SEM. The numbers of animals were shown within parentheses. Contractile and vasodilator responses are shown as a percentage of the reference response to 120 mM KCl and of the isometric contraction induced by phenylephrine (3–10 μM), respectively. *^,#,&,∆^ *p* < 0.05; **^,&&,$$^ *p* < 0.01; ***^,###,&&&,∆∆∆,$$$^ *p* < 0.001 compared to the * WKY, ^∆^ SHR, ^#^ Phe, ^&^ U46619, ^$^ MethAEA, as determined by one-way ANOVA followed by Bonferroni’s multiple comparison test and Student’s *t*-test for unpaired data.

**Table 2:** The influence of AM251 (1 µM) on the vasoconstriction to phenylephrine (Phe) and thromboxane analog U46619 and vasorelaxant effects of acetylcholine (Ach) and sodium nitroprusside (SNP) in the endothelium-intact aortas from normotensive rats: control (WKY) and URB597-treated (WKY + URB), or hypertensive rats: (SHR) and URB597-treated (SHR + URB).

Group	WKY	WKY + URB	SHR	SHR + URB
**Phe**	(7)	(7)	(7)	(7)
pEC_50_	7.3 ± 0.05	7.2 ± 0.03	7.3 ± 0.06	7.5 ± 0.05
R_max_ (%)	82.9 ± 2.9	82.9 ± 4.1	88.8 ± 4.0	92.5 ± 2.8
**Phe + AM251**	(7)	(7)	(7)	(7)
pEC_50_	7.2 ± 0.03	7.4 ± 0.10	7.4 ± 0.06	7.4 ± 0.05
R_max_ (%)	91.3 ± 2.6	83.3 ± 7.1	94.5 ± 5.7	91.6 ± 4.2
**U46619**	(7)	(7)	(7)	(7)
pEC_50_	7.4 ± 0.10	7.4 ± 0.03	8.1 ± 0.05 ***	7.9 ± 0.04
R_max_ (%)	97.2 ± 2.9	106.8 ± 2.5	108.4 ± 3.6	104.7 ± 1.9
**U46619 + AM251**	(7)	(7)	(7)	(7)
pEC_50_	7.7 ± 0.04	7.5 ± 0.06	8.0 ± 0.04	7.8 ± 0.06
R_max_ (%)	98.7 ± 4.0	108.5 ± 2.2	106.4 ± 2.6	107.9 ± 1.1
**Ach**	(6)	(6)	(6)	(6)
pEC_50_	7.2 ± 0.03	6.8 ± 0.02 ***	7.3 ± 0.09	7.7 ± 0.06 ^∆∆∆^
R_max_ (%)	100.0 ± 6.7	96.2 ± 7.0	91.3 ± 4.7	92.6 ± 1.7
**SNP**	(6)	(6)	(6)	(6)
pEC_50_	8.4 ± 0.08	8.1 ± 0.04 *	7.6 ± 0.05 ***	7.7 ± 0.06
R_max_ (%)	113.0 ± 4.8	103.9 ± 2.0	110.0 ± 3.6	100.2 ± 6.9

URB597 1 mg/kg or its vehicle was injected intraperitoneally every 12 h for 14 days. Data are expressed as the mean ± SEM. The numbers of animals were shown within parentheses. Contractile and vasodilator responses are shown as a percentage of the reference response to 120 mM KCl and of the isometric contraction induced by phenylephrine (aortas: hypertensive 0.03 μM and normotensive 0.3 μM rats), respectively. * *p* < 0.05; ***^,∆∆∆^ *p* < 0.001 compared to the * WKY, ^∆^ SHR, as determined by one-way ANOVA followed by Bonferroni’s multiple comparison test and Student’s *t*-test for unpaired data.

## Data Availability

The data presented in this study are available on request from the corresponding author. The data are not publicly available due to privacy.

## Abbreviations

2-AG	2-Arachidonoylglycerol
2-AG-d8	Octadeuterated 2-arachidonoyl glycerol
Ach	Acetylcholine
AEA	Anandamide
AEA-d8	Octadeuterated anandamide
ANOVA	Analysis of variance
CRCs	Concentration-response curves
DMF	N,N-Dimethylformamide
DMSO	Dimethyl sulfoxide
DOCA	Deoxycorticosterone acetate
FAAH	Fatty acid amide hydrolase
H + E	Hematoxylin and eosin
i.p.	Intraperitoneally
Kca	Calcium-dependent potassium channels
LC–MS	Ultrahigh performance liquid chromatography-tandem mass spectrometry
L-NAME	N^G^-nitro-L-arginine methyl ester
MAGL	Monoacylglycerol lipase
MethAEA	Methanandamide
MRM	Multiple reaction monitoring
N.D.	Not determined
Phe	Phenylephrine
RIPA	Radioimmunoprecipitation assay
SBP	Systolic blood pressure
SHR	Spontaneously hypertensive rats
sMA	Small mesenteric G3 arteries
SNP	Sodium nitroprusside
TRPV1	Transient receptor potential vanilloid type 1
TXA_2_	Thromboxane A_2_
UNX	Uninephrectomized normotensive
URB597	3’-(Aminocarbonyl)[1,1’-biphenyl]-3-yl)-cyclohexylcarbamate
WKY	Wistar Kyoto rats
